# Effective Degradation of Gluten and Its Fragments by Gluten-Specific Peptidases: A Review on Application for the Treatment of Patients with Gluten Sensitivity

**DOI:** 10.3390/pharmaceutics13101603

**Published:** 2021-10-02

**Authors:** Yakov E. Dunaevsky, Valeriia F. Tereshchenkova, Mikhail A. Belozersky, Irina Y. Filippova, Brenda Oppert, Elena N. Elpidina

**Affiliations:** 1 A.N. Belozersky Institute of Physico-Chemical Biology, Lomonosov Moscow State University, 119991 Moscow, Russia; dun@belozersky.msu.ru (Y.E.D.); mbeloz@belozersky.msu.ru (M.A.B.); elp@belozersky.msu.ru (E.N.E.); 2 Chemical Faculty, Lomonosov Moscow State University, 119991 Moscow, Russia; v.tereshchenkova@gmail.com (V.F.T.); irfilippoff@yandex.ru (I.Y.F.); 3 USDA Agricultural Research Service, Center for Grain and Animal Health Research, Manhattan, KS 66502, USA

**Keywords:** celiac disease, peptidases, gluten, enzyme therapy, gluten-free diet

## Abstract

To date, there is no effective treatment for celiac disease (CD, gluten enteropathy), an autoimmune disease caused by gluten-containing food. Celiac patients are supported by a strict gluten-free diet (GFD). However, in some cases GFD does not negate gluten-induced symptoms. Many patients with CD, despite following such a diet, retain symptoms of active disease due to high sensitivity even to traces of gluten. In addition, strict adherence to GFD reduces the quality of life of patients, as often it is difficult to maintain in a professional or social environment. Various pharmacological treatments are being developed to complement GFD. One promising treatment is enzyme therapy, involving the intake of peptidases with food to digest immunogenic gluten peptides that are resistant to hydrolysis due to a high prevalence of proline and glutamine amino acids. This narrative review considers the features of the main proline/glutamine-rich proteins of cereals and the conditions that cause the symptoms of CD. In addition, we evaluate information about peptidases from various sources that can effectively break down these proteins and their immunogenic peptides, and analyze data on their activity and preliminary clinical trials. Thus far, the data suggest that enzyme therapy alone is not sufficient for the treatment of CD but can be used as a pharmacological supplement to GFD.

## 1. Introduction

Celiac disease (CD), or gluten enteropathy, is a hereditary predisposed disease, accompanied by the atrophy of the small intestine mucosa, associated malabsorption syndrome, and the development of various deficiency conditions [1,2]. Celiac disease is caused by food containing gluten—the proteins of cereals that are the diet of the majority of the world population. Some immunogenic peptides of gluten proteins formed during digestion, mainly gliadins from wheat, rye, and barley, are resistant to proteolysis by human digestive peptidases [3,4] and cause CD in predisposed people. A strict gluten-free diet (GFD) is still the only means of supporting the health of CD patients, but efficacy is lacking in many that retain symptoms of the disease. In addition, a strict compliance to GFD reduces the quality of life of patients, as it is often difficult to maintain in a professional or social environment. In addition, a gluten-free diet is significantly more expensive than comparable conventional foods [5].

Peptidases that effectively cleave these difficult-to-hydrolyze peptides can be used as an enzyme therapy for patients with CD. The gluten proteins of cereals, and especially immunogenic peptides formed from them, are rich in proline, which is the only imino acid among the twenty natural proteinogenic amino acids. The presence of a proline residue in the polypeptide chain changes its conformation, preventing the degradation of proline-rich proteins by broad-spectrum peptidases. In nature, hydrolysis of proline-containing proteins is carried out mainly by a special group of proteolytic enzymes—proline-specific peptidases (PSPs). Due to their unique specificity, PSPs are involved in the “fine” regulation of various metabolic processes, cell differentiation and maturation, the processing of biologically active peptides and proteins, and the formation of an immune response. Therefore, the research regarding PSPs has been primarily directed to the study of their regulatory function with a strong medical focus, as PSPs are involved in a wide variety of diseases, such as diabetes, cancer, Alzheimer’s and Parkinson’s diseases, hypertension, and neuropsychiatric diseases [6]. The ability of PSPs to neutralize the immunogenic potential of proline-rich gluten peptides is a way to exploit their unique specificity and presents an attractive option for patients with CD. 

The immunogenic peptides of gluten are not only rich in proline but also glutamine. Therefore, peptidases are needed with activity towards glutamine that can either independently or in combination with PSPs partially or completely eliminate the immunogenicity of these peptides. Oral enzyme therapy using such gluten-destroying peptidases is a promising therapeutic approach. This narrative review describes the features and characteristics of the main proline/glutamine-rich proteins of cereals, analyzes peptidases from various sources that can effectively break down these proteins, and discusses their potential to produce gluten-free products, including the problems in incorporating these enzymes in therapies for CD.

## 2. Prolamins 

Gluten proteins are the main storage proteins of cereal seeds, such as wheat, barley, and rye. Initially, the classification of these proteins was based on their solubility, and fractions included those that are alcohol-soluble or alcohol-insoluble [7]. Alcohol-soluble proteins were called prolamins according to their amino acid composition—prolamins are rich in proline (up to 30%) and glutamine (up to 50%) [7,8,9,10]. In wheat, prolamins are called gliadins, in barley—hordeins, and in rye—secalins. Alcohol-insoluble fractions were called glutelins, and in wheat—glutenins. Initially, prolamins and glutelins were assigned to different groups of proteins, but later it turned out that many glutelins are related to prolamins in their primary structure. The insolubility of glutelins in alcohol is due to the fact that they form high-molecular polymers stabilized by disulfide bonds between individual polypeptides. Since many glutelins are structurally related to prolamins, and some reduced polymer subunits are alcohol soluble and rich in proline and glutamine, they are now also considered prolamins [7,8,9]. 

Gliadins and glutenins in wheat grains are the main components of gluten. It is gluten that provides wheat with the required dough-forming and baking qualities, as well as most of the unique taste of wheat-based products. Gluten proteins are extremely heterogeneous. They can be divided into three main groups: high-molecular-weight prolamins (high-molecular-weight gluten subunits), S-poor prolamins (ω-gliadins), and S-rich prolamins, which include gliadins and low-molecular-weight prolamins (low-molecular-weight gluten subunits). Gliadins are divided into four discrete electrophoretic fractions, α-, β-, γ-, and ω-gliadins, which differ both in molecular mass (M_r_ = 31,000−42,000 Da) and in amino acid sequences [8,11].

There are individual sites or domains in the structure of prolamins that have both a diverse amino acid composition and possibly different origins, and amino acid sequences consisting either of repeating blocks based on one or more short peptide motifs, or enriched with certain amino acid residues, such as Gln, Pro, or Met [8]. The peptide bonds formed by these amino acids, primarily the cyclic imino acid residue Pro which disrupts the structure of the protein helix, are not hydrolyzed by most known peptidases since they do not correspond to their specificity. That is, the enzymatic hydrolysis of prolamins can be performed efficiently only by a limited group of peptidases. Meanwhile, in nature, these storage proteins are hydrolyzed and absorbed both in plant seeds during germination, and in humans and farm animals that feed on grain products. However, in genetically predisposed people, some peptides rich in proline and glutamine formed during the hydrolysis of prolamines cause CD [1,2,12,13].

## 3. Celiac Disease (CD)

Celiac disease or gluten-sensitive enteropathy is a classic autoimmune disease, because it is characterized by tissue immunogenic inflammation and occurs in individuals with a specific set of HLA genes, namely those people whose genome contains certain alleles of the T-cell-specific immune response genes—HLA-DQ2 and HLA-DQ8, which are part of the HLA-DR3 genotype. HLA-DQ2 and HLA-DQ8 molecules have a high affinity for deamidated gliadin peptides, and in complex with tissue transglutaminase (TG2), present them to immune cells. TG2 deamidates gliadin peptides to form negatively charged deamidated peptides, leading to enhanced binding to HLA-DQ2 or HLA-DQ8 and subsequent presentation to the immune system, resulting in strong immunogenic inflammation in the intestinal wall. The T-cell-specific immune response is to gliadins, their deamidated fragments (peptides), TG2, and connective tissue proteins that are part of the endomysium and reticulin. Manifestations of autoimmune reaction are the destruction of the small intestinal mucosa and malabsorption of nutrients [14].

Proline- and glutamine-rich peptides derived from α-gliadins (a low-molecular-weight variety of gliadins) and γ-gliadins (cysteine-rich gliadins stabilized by disulfide bonds) containing more than nine amino acid residues are toxic to predisposed people [3,4,15]. For example, the 33-mer α-2 gliadin peptide LQLQPFPQPQLPYPQPQLPYPQPQLPYPQPQPF is a substrate for a TG2 enzyme that modifies glutamine residues to promote pathogenesis. Another 26-mer peptide, FLQPQQPFPQQPQQPYPQQPQQPFPQ, is formed from γ-5 gliadin. Shorter immunogenic proline- and glutamine-rich peptides containing 10–20 amino acid residues (Table 1) often are used for model studies [16]. However, it is the 33- and 26-mer peptides, derived from α- and γ-gliadins, respectively, that are particularly strong activators of T-cells and therefore strongly correlate with the onset and development of CD [3,4].

The intensity of the inflammatory process in CD varies from an increased content of intraepithelial lymphocytes in the epithelium of the villi of the small intestine to atrophy of the mucous membrane. Due to the complexity of the diagnosis, which was previously based on the results of a biopsy, CD was considered a rare disease that was mostly found in Europeans and manifested during the first years of life. Later, sensitive and specific serological tests permitted estimates of the actual number of patients [17]. Screening studies have shown that this disease is not age-related, can occur at any time, and is much more common than previously thought, namely 1% of the world’s population. In most patients, CD occurs with mild symptoms or has atypical clinical manifestations. A persistent epidemiological pattern is the steady increase in gluten intolerance in humans [18,19]. The reasons for this can be the following factors: the increase in gluten consumption worldwide; early introduction of complementary foods containing cereals in children of the first year of life against the background of a decrease in the duration of breastfeeding; the emergence of new varieties of wheat with a high content of gluten; and accelerated methods in the production of bakery products (reducing the fermentation period) that increase the content of toxic gluten peptides [20]. Malabsorption is the classic manifestation of CD. At the same time, the following symptoms are observed: chronic diarrhea, flatulence, weight loss, and vitamin and microelement deficiencies. Over time, there is a high risk of developing cancer and other autoimmune diseases, as well as nervous disorders [21,22,23].

Currently, there is no cure for CD. A strict gluten-free diet (GFD) is the only effective way to maintain the health of CD patients. In most patients with gluten sensitivity, the introduction of GFD leads to at least partial healing of the duodenal mucosa, improvement of most symptoms associated with gluten consumption, and a decrease in the titers of specific antibodies in gluten disease. However, in many patients, even with long-term strict adherence to GFD, symptoms may persist, including inflammatory and architectural changes in the small intestine mucosa and positive antibody levels [24,25]. A number of factors may contribute to an incomplete response to a GFD. It is difficult to avoid cross-contamination during food production because gluten is widely used in the food industry. Food labeling may be inaccurate, misleading, or incorrect. In a double-blind clinical trial, patients with CD in remission who were given 50 mg of gluten daily experienced a 20% reduction in villus height/crypt depth compared to a daily placebo or 10 mg of gluten [26]. This indicates that even traces of gluten can cause chronic mucosal damage. The acceptable (safe) limit of gluten may vary from patient to patient and may correspond to 10–100 mg per day, even though a slice of wheat bread contains approximately 3–4 g of gluten [27]. Sticking to such a strict diet is difficult; generally, it is more expensive, less accessible, severely restricts food choice, may result in products with off-taste, and may lead to asocialized individuals (especially in adolescents) and depressive states [5]. Moreover, there is a lack of vitamins and minerals, as well as a tendency to anemia and osteoporosis, in patients on GFDs. In most cases, unintended gluten exposure can occur in patients as a result of the consumption of 10–1000 mg of “hidden” gluten contained in common food ingredients such as sauces, salad dressings, food starches, malt extract thickeners, and other flavors, and sometimes simply as a result of cross-contamination during cooking. Thus, the complete elimination of gluten is, at best, a difficult task. Despite attempts to adhere to GFD, long-term treatment of patients with gluten disease often results in severe atrophy of the villi [25]. It is possible that many patients are inadvertently consuming hundreds of milligrams (or more) of gluten per day. Therefore, there is a need to develop a non-dietary (pharmacological) therapy that would either supplement or replace GFD and neutralize up to 1 g of gluten while the food is still in the stomach.

Various therapeutic strategies are being developed to combat CD. Enzyme therapy is especially promising, as a supplement to food in the form of a peptidase preparation that efficiently degrades prolamins peptides [28]. This approach is based on a direct effect on the pathogenic substance, namely, uncleaved peptides with a large number of proline and glutamine residues that are not digested by typical stomach enzymes.

## 4. Peptidases that Effectively Hydrolyze Prolamins and Their Immunogenic (Toxic) Peptides

Since immunogenic gliadin peptides are rich in proline residues, PSPs can be used to cleave bonds formed by the Pro residue in proteins and peptides [28]. PSPs characterized thus far have different substrate specificity (Table 2). Most PSPs are exopeptidases: dipeptidyl peptidase (DPP) 2, DPP 4, DPP 8, DPP 9, prolyl carboxy peptidase (PRCP), aminopeptidase P (APP) 1, APP2, APP3, and prolidase. PSP endopeptidases, prolyl oligo peptidases (POP) and prolyl endo peptidases (PEP), usually have higher efficacy. Fibroblast activation protein (FAP) possesses both exo- and endopeptidase activity. All PSPs belong to one of two classes of peptidases—either serine or metallopeptidases. PSPs that are effective in detoxifying the immunotoxic prolamin peptides are found in various organisms belonging to different kingdoms of wildlife.

In addition to proline, the other most common amino acid residue in cereal prolamins is glutamine, so that peptidases with specificity toward this residue also are needed. The activity of cysteine post-glutamine cleaving peptidases (PGP) was detected in the larval midgut tissue of the Tenebrionidae beetles *Tenebrio molitor* and *Tribolium castaneum* using highly specific peptide substrates Z-Ala-Ala-Gln-pNA, Glp-Phe-Gln-pNA, and Glp-Phe-Gln-AMC, where Z is benzyloxycarbonyl, Glp—pyroglutamyl, pNa—*p*-nitroanilide, and AMC—4-amino-7-methylcoumaride [29,30,31]. Post-glutamine cleaving activity has also been found in studies of the hydrolysis of proline- and glutamine-rich immunogenic peptides by subtilisin-like peptidases of bacteria [32] and cysteine peptidases of plants [33,34].

### 4.1. Hydrolysis of Gluten Proteins and Their Toxic Peptides by Bacterial Peptidases

A study of the hydrolytic properties of bacterial POP from *Myxococcus xanthus* (MX), *Sphingomonas capsulata* (SC), and *Flavobacterium meningosepticum* (FM) on two gliadin peptides that play a key role in the development of CD (from α-2 gliadin) revealed significant differences in the specificity of enzymes associated with the length of the substrate [35,36,37,38]. SC-POP effectively hydrolyzed the shorter substrate PQPQLPYPQPQLP but had low activity against longer LQLQPFPQPQLPYPQPQLPYPQPQLPYPQPQPF. In contrast, FM-POP and MX-POP cleaved both substrates, although FM-POP had greater activity with the longer substrate than MX-POP. Analysis of the hydrolysis products showed that SC-POP cleaved both Pro-Gln and Pro-Tyr bonds (PQPQLP↓YP↓QPQLP), FM-POP cleaved the Pro-Gln bond better (PQPQLPYP↓QPQLP), and MX-POP preferred the Pro-Tyr site (PQPQLP↓YPQPQLP). MX-POP formed short fragments of 4–5 amino acid residues with LQLQPFPQPQLPYPQPQLPYPQPQLPYPQPQPF, while FM-POP formed long intermediates, mainly due to the preferred cleavage of the central Pro-Gln bond of the substrate. Thus, there were clear differences in the specificity of POPs from different organisms, but their high hydrolytic activity with both immunogenic gliadin peptides make them potential candidates for enzyme therapy of CD [35]. Testing the therapeutic value of POP requires a long-term study of CD patients who receive controlled amounts of POP-processed and unprocessed gluten.

**Figure 1:**
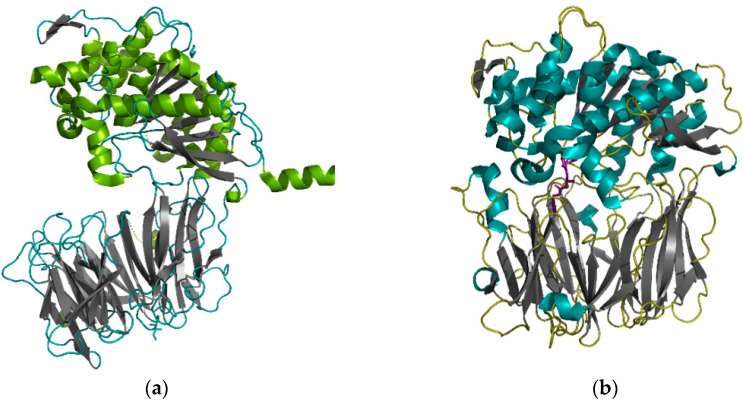
(**a**) Structure of SC-POP (PDB 1YR2) with a resolution of 1.8 Å. The α-helices of the catalytic domain are green, β-sheets of the β-propeller domain are gray. (**b**) Structure of MX-POP bound with Z-Ala-prolinal inhibitor (PDB 2BKL). The α-helices of the catalytic domain are teal, β-sheets of the β-propeller domain are gray. Inhibitor Z-Ala-prolinal is shown as sticks and is purple. Structures are visualized using PyMOL (https://pymol.org/2/, accessed on 25 May 2021) (The PyMOL Molecular Graphics System, Version 1.2r3pre, Schrödinger, LLC, New York, NY, USA).

SC- and MX-POP were obtained as recombinant proteins [39]. The tertiary structures of the enzymes were determined by X-ray diffraction: the open structure of SC-POP with a resolution of 1.8 Å (Figure 1A), and the structure of MX-POP in a complex with the Z-Ala-prolinal inhibitor with a resolution of 1.5 Å (Figure 1B). Bacterial POPs consisted of an α-catalytic domain with a catalytic triad of Ser, Asp, and His, and a β-propeller domain responsible for substrate specificity. Their active sites lay near the contact boundary between these two domains, so mutagenesis at the interdomain interface could be used to change protein dynamics, which in turn would affect the change in substrate specificity.

MX-, SC-, and FM-POP cannot hydrolyze whole gliadins. However, they can hydrolyze proline-rich peptides that are formed by the action of human digestive peptidases. The length of such peptides is from 9 to 33 amino acid residues, that is, sufficient for the potential hydrolysis by these POP enzymes. Thus, the treatment of gluten, pre-hydrolyzed with a mixture of pepsin-trypsin-chymotrypsin, with recombinant FM-POP reduced the amount of potentially immunogenic peptides in vitro and ex vivo, and also avoided the development of fat and carbohydrate malabsorption in most patients with CD who developed the condition after two weeks of provocative gluten intake [38]. The difficulties in using these enzymes are associated with their optimal pH, which lies in the range of 7.0–8.5, so they cannot function in the stomach at acidic pH values, and, moreover, they are unstable due to their degradation by pepsin [40]. An enteric coating (decomposing in the intestine) on a gelatin capsule containing the enzyme preparation can improve the protection of the enzyme from the gastric environment (>1 h in 0.01 M HCl, pH 2 with pepsin), but may lead to a delay in the release of the POP enzyme in the duodenum (pH 6.0 in the presence of trypsin and chymotrypsin) [36]. Therefore, it is necessary to optimize the amount and application of the enteric coating for successful delivery of the enzyme preparation (protection and release). The possibility of the therapeutic use of enzymes requires more in-depth analysis of their specificity for other immunogenic gluten peptides [16], as well as determining their stability in the presence of bile salts and other substances usually found in the intestine. Another important aspect to consider is the time it takes for POP to digest the peptides, because intact peptides or immunogenic fragments can cross the small intestine mucosa before they undergo complete hydrolysis, and peptidase activity will be determined by the amount of POP. The theoretical dose of POP that will be needed for a sufficiently complete and rapid digestion of gluten present in normal food is difficult to calculate. It is a dynamic system, and the calculation of the relative rates of peptide transport and corresponding degradation of POP as it passes through the gastrointestinal system will be one of the key points in planning the future use of POP for enzyme therapy [41]. 

A recombinant strain of *Lactobacillus casei*, which produces the secreted activity of MX-POP, has been proposed for the delivery of POP to the intestinal environment [42]. The secreted enzyme is able to destroy the immunotoxic 33-mer gliadin peptide, which plays a key role in the pathogenesis of CD. This strain survives a simulated gastrointestinal transit maintaining the ability to produce and secrete MX-POP and thus may be a good system for delivering MX-POP to the duodenum of patients with CD. The authors believe that the genetically engineered food product may be useful as a vector for the production of POP in situ in the upper small intestine of CD patients after additional research and clinical trials.

A recombinant form of a new POP with a molecular mass of 77 kDa from the thermostable bacteria *Sphaerobacter thermophiles* was able to hydrolyze bonds after proline residues in the toxic peptide of α-gliadin, LGQQQP↓FPP↓QQP↓Y, PQPQLPYPQPQLP↓Y, and SQQQFP↓QPQQP↓FP↓QQP of γ-hordein. The enzyme was stable in the pH range of 5.0–8.0 with an optimum activity at pH 6.6. This POP was thermally stable with temperature-optimum activity near 63 °C [43]. Due to these features (neutral pH optimum and high temperature optimum), this POP was not proposed as an enzymatic treatment of CD, but instead can be useful for the production of gluten-free beverages and food. The addition of the enzyme to barley malt reduced the concentration of gluten. Thus, the *S. thermophiles* POP can be used for high-temperature mashing of barley malt in the brewing process [43].

The traditional process of producing different varieties of wheat and rye bread, as well as improving its quality, is the fermentation of sourdough consisting of a symbiotic culture of yeast and lactic acid bacteria (LAB) growing in a mixture of flour and water. Sourdough fermentation with LAB is used to make the flour suitable for baking, to control the development of characteristic flavor components, to achieve a yeast leaven of the dough, to suppress undesirable fermentation by other bacteria and yeast, and to increase the shelf life of bread [44]. During the fermentation of the dough, proteolysis by LAB releases small peptides and free amino acids, which are important for the rapid growth of microbes and acidification, as well as being precursors for the development of the flavor of yeast baked goods. In addition, this proteolytic activity can be used as a tool to reduce the amount of certain allergenic compounds derived from gluten (gliadin peptides), which are often present in wheat baked goods and cause CD. A study of the hydrolysis of α-gliadin fragments by peptidases from sourdough of *Lactobacillus plantarum* and *Pediococcus pentosaceus* strains, among which proline-specific peptidases are prominent, showed that *L. plantarum* strains hydrolyzed more than 60% of α-gliadin peptides involved in the immune response in CD patients, corresponding to fragments 31–43 (LGQQQPFPPQQPY) and 62–75 (PQPQLPYPQPQSFP). None of the lactic acid bacteria strains cleaved fragment 57–89 (LQLQPFPQPQLPYPQPQLPYPQPQLPYPQPQPF), but a mixture of *L. plantarum* CRL 775 and *P. pentosaceus* CRL 792 hydrolyzed this peptide by 57% in 8 h [45,46]. Further studies are required to determine the optimal dose of LAB and their combinations, and the time for fermentation to determine the practical application of these results.

Screening of 12 strains of LAB and yeast, based on their ability to hydrolyze wheat proteins and ferment dough, found that no unique strain of the LAB or yeast can completely degrade wheat allergens [47]. However, *Pediococcus acidilactici* XZ 31, *Torulaspora delbrueckii* JM1, and *Saccharomyces cerevisiae* JM4 demonstrated superiority over other strains in their ability to ferment dough and reduce the allergenicity of wheat products. Further in vivo testing of the anti-allergenic potential is required to confirm that selected LAB and yeast strains can be considered as an effective starter culture for the preparation of hypoallergenic wheat products.

Controlled proteolysis in wheat dough also has been suggested to reduce the level of gliadins to such an extent that the products obtained are tolerated by patients with CD. However, the expected difficulty from a technological point of view is related to the suitability of such wheat flour to provide the desired quality of bread, taking into account the complete (or very deep) degradation of gluten. Products with a reduced gluten content and an extended fermentation time may not be suitable for bread production. Low-gluten doughs can only be used as improvers for baking gluten-free bread with lower quality compared to common (wheat) products [48,49] or used in the production of certain products, such as cookies, cakes, and pastries.

Hydrolysis of the immunogenic 33-mer proline-rich peptide (see Table 1) was performed with peptidases from *Lactobacillus sanfranciscensis*, *L. alimentarius*, *L. brevis*, and *L. hilgardii* [50]. Various peptidases were isolated from these LAB: PepN—aminopeptidase, PepI—proliniminopeptidase, PepX—X-prolyldipeptidylaminopeptidase (which shares substrate specificity with DPP 4), PepO—endopeptidase, POP—prolyloligopeptidase, PepT—tripeptidase, PepV—dipeptidase, PepQ—prolidase, and PepR—prolinase. Among the isolated PSPs, only POP hydrolyzed the Pro-Phe bond in the 33-mer, resulting in two peptides, one of which is immunogenic. However, to achieve complete hydrolysis of the 33-mer to free amino acids, it was necessary to use a combination of isolated peptidases PepN, PepX, PepO, POP, PepT, PepV, and PepQ. It was concluded that LAB peptidases can be safely used to modify the diet of CD patients because of their ability to hydrolyze proline-rich peptides of gluten from varieties of *Triticum turgidum* L.

Screening of gliadin-cleaving proteolytic activity among 20 *Lactobacillus* strains showed that the most active strain, *L. casei*, was able to hydrolyze the 33-mer immunogenic peptide of α-gliadin by 82% in 8 h, and completely in 12 h [51]. Other authors selected 11 strains of LAB, belonging to the species *L. curvatus*, *Pediococcus acidilactici*, *P. pentosaceus, L. coryniformis, Weissella cibaria, L. plantarum,* and *L. helveticus,* [52], as well as probiotic strains of *Bifidobacterium bifidum*, *B. longum*, *B. breve*, and *B. animalis* [53]. A probiotic mixture (two strains of lactobacilli and three strains of bifidobacteria) reduced the toxicity of gliadin fragments remaining after peptic-tryptic digestion by degrading immunodominant gliadin peptides [54].

A mixture of *L. acidophilus* 5e2 and *Aspergillus niger* peptidases was able to hydrolyze both gliadins and the toxic peptides. The concentrations of the immunogenic peptides LGQQQPFPPQQPY and PQPQLPYPQPQLP decreased by 126 and 31 times, respectively, during 3 h of incubation at pH 4.0 and 37 °C. The authors believe that the technology does not guarantee the complete breakdown of cereal proteins but can be used for faster and more efficient degradation of immunoreactive peptides than traditional processes [55].

A good source of peptidases that can effectively hydrolyze gliadins and their proline- and glutamine-rich peptides was found in bacteria from human salivary fluid [56,57,58]. Using various synthetic substrates, a suspension containing bacteria from salivary fluid and oral plaque effectively hydrolyzed Z-Tyr-Pro-Gln-pNA after the glutamine residue. The authors also isolated and identified bacterial strains of *Rothia aeria* and *R. mucilaginosa* that effectively hydrolyzed gliadins and their toxic peptides at significantly shorter times compared to the initial total suspension [59,60]. *R. aeria* effectively hydrolyzed the main fraction of gliadins (in the region of 37 kDa) in a 30 min incubation, and after 2 h the band of the target fraction was not detected. The enzyme from *R. mucilaginosa* was subsequently isolated and identified as belonging to the subtilisin family S8 of peptidases. The isolated subtilisin-like enzyme from *R. mucilaginosa* was highly effective in hydrolysis and neutralization of gluten epitopes involved in the provocation of CD [32]. Thus, subtilisins represent another class of enzymes with great potential for enzymatic therapy of CD. 

However, the problem is that subtilisins are weakly active in the acidic conditions of the stomach, and thus instability and autodegradation are the main obstacles for their therapeutic use. There are two methods of pharmaceutical modification to protect and maintain the activity of subtilisin (Sub): covalent or non-covalent attachment of polyethylene glycol (PEG) to the protected protein molecule (PEGylation), and coating with polymer microparticles that partially resist enteral degradation and provide targeted delivery of the pharmacological agent based on copolymers of polylactic and glycolic acids (PLGA, microencapsulation). The PEGylation of subtilisin by attaching methoxypolyethylene glycol (mPEG) protected the enzyme from autolysis at neutral pH [61]. The PEGylated enzyme (Sub-mPEG) was further encapsulated by PLGA. Microencapsulated Sub-mPEG-PLGA showed significantly increased protection against acid exposure in vitro. In vivo, the immunogenic gluten epitopes in the stomach of mice fed a diet containing Sub-mPEG-PLGA decreased by 60% compared to 32% in mice fed a diet containing unmodified Sub. These results show that pharmaceutical modification can protect Sub from self-digestion, as well as from acid inactivation, which undoubtedly makes the enzyme more effective for use in vivo. Such modifications can be applied to other enzymes that effectively hydrolyze immunogenic peptides but are not stable enough under working conditions.

Comparison of salivary enzyme activity profiles from the oral cavity of healthy people and patients with CD showed that the activity of salivary glutenases was higher in patients with CD [62]. The oral microbiomes of patients with CD differed significantly from healthy ones, with higher levels of salivary lactobacilli in CD individuals, which may partially explain the observed increased gluten-cleaving activity. This correlation between oral and intestinal microbiomes with CD requires further in-depth study. However, it suggests that the activity of salivary microbial glutenase is higher in patients with CD and may affect the processing of gluten and immunogenic epitopes before entering the stomach and small intestine. Considering that the rates of degradation of the gluten substrate in saliva are relatively low, enzymes from the endogenous microbiome of the oral cavity are likely unable to fully digest food containing gluten. In this case, the activity of microbial enzymes in the oral cavity may contribute to the release of larger amounts of immunogenic gluten peptides. Consequently, an increase in incomplete digestion by the endogenous microbiomes of the oral cavity and duodenum may lead to an increase in transepithelial transport of active immunogenic gluten epitopes and, thereby, promote CD activation. This was confirmed in the mouse CD model, where as a result of partial digestion of gluten by Pseudomonas aeruginosa, smaller peptides were then more easily transported through the mouse intestinal barrier and fueled the activity of the disease [63,64]. Thus, the increase in incomplete digestion, which may be observed in the oral cavity of patients with CD, leads not to a decrease, but to an increase in the immunogenicity of gluten, due to the formation of more peptides with immunostimulating potential early in the digestion of food.

Most research described in this review is directed to the search and study of peptidases capable of cleaving gliadins and their toxic peptides, based on their ability to hydrolyze the bonds formed by proline or glutamine residues. Gordon et al. [65] took a different approach. Using computer simulations, they selected a peptidase with high cleavage activity at low pH, satisfying specific requirements for enzymatic affinity. As a result, one enzyme, Kumamolysin-AS (KumaWT), from the acidophilic bacterium *Alicyclobacillus sendaiensis* was selected. KumaWT is an endopeptidase with high activity at acidic pH values and with specificity for the dipeptide motifs PR and PK, close to the PQ motif found in toxic gliadin peptides. KumaWT is a serine endopeptidase with a catalytic Ser-Glu-Asp triad, different from the Ser-His-Asp triad of classical serine peptidases. KumaWT is active at low pH values with a maximum in the range from 2.0 to 4.0 due to a glutamic acid residue with a pKa of 4.1, and specifically recognizes the PR and PK motifs. Spatial structure analysis showed that the S1 binding subsite responsible for the coordination of amino acid residues in the P1 position of the substrate (Arg, Lys) includes Asp358 and Asp368. The proline residue in the P2 position of the substrate binds in the S2 subsite represented by Trp318. Possible mutations sites that may enhance the desired oligopeptide specificity of the enzyme were identified. As a result, the proposed modified enzyme, KumaMax, contained seven mutations: Val119Asp, Ser262Lys, Asn291Asp, Asp293Thr, Gly319Ser, Asp358Gly, and Asp368His, and exhibited maximum activity on a substrate including the PQPQLP fragment. Of these, the residues Gly319Ser, Asp358Gly, and Asp368His formed new hydrogen bonds with Gln at the P1 position. The simulated KumaMax enzyme hydrolyzed the substrate containing the PQPQLP fragment 116 times more efficiently than the original KumaWT enzyme. The authors demonstrated the ability of KumaMax to cleave the immunogenic peptide QLQPFPQPQLPY of α9-gliadin.

The stability of KumaMax in the presence of the main gastrointestinal peptidases was investigated [65]. The enzyme was incubated for 30 min with pepsin at pH 4.0 and trypsin at pH 7.0, and KumaMax was stable with both enzymes. For comparison, the POP from *S. capsulate* (SC-POP) was degraded by both enzymes, and an endopeptidase from barley (EP-B2) was stable only in the presence of pepsin under gastric digestion conditions and underwent significant proteolysis under the trypsin treatment.

Later, a computer redesign of the active site of KumaMax led to the drug Kuma030, which specifically recognizes tripeptide sequences (PQL or PQQ) characteristic of the immunogenic regions of gliadins, as well as homologous proteins of barley and rye [66]. Treatment of gliadins with Kuma030 eliminated the T-cell response to gliadins. Kuma030 was able to degrade more than 99% of the immunogenic gliadins fraction in laboratory-modeled gastric digestion in a physiologically significant time frame, to a level below the toxic threshold for patients with CD, suggesting great potential for this enzyme as an oral therapeutic agent. However, there are no data on the results of clinical trials of this enzyme, and it is not yet commercially available.

Another well-studied enzyme that breaks down gluten is latiglutenase (formerly known as ALV003), a mixture of two recombinant proteases that are in phases 1 and 2 of clinical trials [67]. One of these peptidases is a modified recombinant version of prolyloligopeptidase from the bacterium *Sphingomonas capsulata* (SC-POP) with PSP cleavage activity; the other is a cysteine peptidase from barley (EP-B2), which has post-glutamine cleavage activity. Together, these enzymes in vitro break down gluten significantly faster than any of the enzymes alone. This drug significantly reduced the mucosal damage found in the placebo group (the ratio of villi height to crypt depth, the number of intraepithelial lymphocytes) when administered together with 2 g of gluten for 6 weeks in patients with CD [68]. At the same time, no statistically significant effects on serum levels of TG2-IgA, antibodies to deamidated gliadin peptides, or on symptoms were reported. When tested in a large-dose-range study involving nearly 500 CD patients, there were no differences in histological pattern or evaluation of serological markers between latiglutenase and placebo [69]. However, there was a clinically and statistically significant improvement in the frequency and severity of abdominal pain, and a reduction in bloating and fatigue. This observation may indicate that latiglutenase treatment affects symptoms before clinically significant effects on serological and histological endpoints appear. Studies by Syage et al. [70,71] also indicated an improvement in symptoms in patients identified as seropositive (positive for serum IgA antibodies to TG2 and/or IgA or IgG antibodies to deaminated gliadin peptides) after taking latiglutenase with a meal compared to placebo. The authors also speculated that the reason for symptom improvement without histological improvement may be related to the time intervals required for histological improvement and improvement of symptoms, but also the poor relationship between the severity of symptoms and the degree of villous atrophy and gluten intake.

ImmunogenX, a clinical biotherapy company, has published the results of phase 2 trials of latiglutenase [72]. These results demonstrated that latiglutenase was safe and effective in reducing the symptoms of seropositive CD patients on a gluten-free diet. Significant improvements in both symptom severity and quality of life were observed in seropositive patients who had CD-specific antibodies in their blood, but not in seronegative patients who lacked CD markers in their blood but still had intestinal damage and had one or more HLA genes for CD. A total number of 398 CD patients completed the 12-week study, taking either an oral dose of latiglutenase or a placebo three times a day. At 0, 6, and 12 weeks of treatment, patients completed a daily symptom diary and numerous quality of life questionnaires. Patients with seropositive CD experienced a reduction in abdominal pain, bloating, fatigue, and constipation. More symptomatic patients experienced a greater reduction in these symptoms. The symptoms of nausea and diarrhea, however, did not significantly improve. Thus, the results indicate that a statistically significant and dose-dependent improvement with latiglutenase is observed in seropositive, but not in seronegative patients with CD [71,73].

In summary, although latiglutenase does not induce healing of mucous membranes and does not show clear benefits in seropositive patients, it does reduce the severity and frequency of key symptoms in CD patients on GFD and improve their quality of life, and this makes latiglutenase potentially promising for future treatment of CD.

### 4.2. Hydrolysis of Gluten Proteins and Their Toxic Peptides by Fungal Peptidases

The effect of PEP from the *Aspergillus niger* fungus (AN-PEP) on gliadins has been the most well studied [74]. The amino acid sequence of AN-PEP has 526 residues and a molecular mass of about 66 kDa, with a 22 amino acid signal peptide and a propeptide containing 19 residues. Comparison with other PEP sequences indicated that AN-PEP has a significant homology with PRCP and DPP 2, assigning this peptidase to the S28 family of serine peptidases.

AN-PEP cleaved whole gliadins, as well as their peptic-tryptic hydrolysates, with high efficiency. AN-PEP hydrolyzed gliadin fragments much more efficiently compared to FM-POP from *F. meningosepticum*: the rate of hydrolysis by AN-PEP was 60 times higher than FM-POP. At the same time, the data indicate that the two enzymes are very similar in their action on gluten epitopes with different structures. Both AN-PEP and FM-POP have post-proline cleaving activity, and both more effectively hydrolyze peptide bonds located in the middle of the molecule, rather than near the N- and C-terminus. In some cases, the hydrolysis products of gliadin peptides are the same, but in general, FM-POP cleaves the peptides into a larger number of fragments. A notable difference is that FM-POP has the greatest activity at pH 6.0–8.0, while AN-PEP is stable at acidic pH values (pH 2–8, optimal activity pH 4–5)—that is, in conditions similar to those characteristic of the human gastrointestinal tract—and is also resistant to degradation by pepsin. Therefore, AN-PEP is a better potential candidate for the treatment of CD than FM-POP. The ability of AN-PEP to degrade gluten into non-immunogenic peptides was demonstrated in in vitro and in vivo studies [74,75,76]. AN-PEP can effectively degrade gluten in the stomach of individuals when added to food administered intragastrically [76,77]. Despite these promising results, the authors are uncertain if AN-PEP can protect gluten-sensitive people who consume gluten. An oral enzyme cannot replace a gluten-free diet, but it may be useful as a digestive aid to facilitate the digestion of “hidden” gluten. In other words, the enzyme can protect against daily unintentional ingestion of gluten, as well as during social events, meetings, or travel. 

In 2017, a double-randomized placebo-controlled clinical trial of a drug created on the basis of AN-PEP by DSM Nutritional Products (Kaiseraugst, Switzerland) and produced by Aenova Group (Munich, Germany) was conducted [77]. The study involved 18 gluten-sensitive individuals. Participants ate a meal containing 0.5 g of gluten and two high- or low-dose AN-PEP or placebo tablets. After 180 min, the contents of the stomach and intestines were collected, and the remaining amount of gluten was evaluated. The level of gluten was decreased in the stomach by 8-fold at a high dose of AN-PEP (relative to the placebo taken as a control) and 7-fold at a low dose, and in the intestine there was a decrease of 2.2-fold and 1.9-fold, respectively. Thus, AN-PEP significantly reduced the amount of gluten in gastric digestion even before the contents entered the intestine. The limitations of the study were that the effect of AN-PEP on the improvement of symptoms was not evaluated, and no data were provided on the histological analysis of the results of the use of AN-PEP. A study of the effectiveness of AN-PEP in the decomposition of a small amount of gluten (about 0.5 g) showed that AN-PEP can be successfully used as an oral dietary supplement by patients with gluten intolerance to support digestion in case of accidental consumption. The mechanism of gluten decomposition in the stomach by ingestion of an enzyme likely will not differ in patients with CD and healthy people. However, the composition of the food significantly affected the amount of AN-PEP required for the complete digestion of gluten, which may make it difficult to use AN-PEP as a complete replacement for GFD [78].

Unfortunately, studies of the effect of gluten-cleaving fungal enzymes on symptoms and biomarkers in patients with CD in randomized placebo-controlled trials are ambiguous. Tack et al. [79] randomized 14 patients with CD to receive 7 g of gluten daily with AN-PEP or a placebo for 2 weeks. However, such a relatively high amount of gluten did not lead to clinical deterioration, even in the group of patients receiving placebo, so it was impossible to show the effect of the enzyme on the change in clinical markers and symptoms. Quite often, patients with CD do not show symptoms when consuming gluten, especially after a long period of abstinence, even if immunological reactions and damage of the small intestine tissue have occurred [80].

Tolerase G, a dietary supplement offered by DSM (Kaiseraugst, Switzerland) also containing AN-PEP, is currently positioned as an ideal product for gluten-sensitive people trying to follow a GFD, as it has the potential to digest hidden or residual gluten present in a wide range of products [81]. However, this product is not suitable for people with CD or gluten intolerance. Thus, Tolerase G is not an effective treatment for CD, since it does not completely break down gluten, and the resulting accumulation of gluten peptides in the duodenum has not been determined. As mentioned above, AN-PEP is able to degrade gluten proteins under acidic conditions in vitro and can apparently be considered as an oral supplement to reduce gluten exposure. However, the uses of glutenases, including AN-PEP, are limited because of incomplete degradation of gluten [82]. 

PEP from the basidiomycete *Flammulina velutipes* (FV-PEP) is similar in specificity to AN-PEP, although the sequence similarity between them was only 39% [83]. The isolated enzyme turned out to be a serine peptidase of the S28 family, with a molecular mass of 50 kDa and the activity maximum at pH 4.5 and 45 °C. FV-PEP degraded the antigenic epitopes of α-gliadin that provoke CD. In the future, this enzyme may become an effective tool for processing wheat and other gluten-containing cereals into more easily digestible products for celiac patients.

Two PSPs from the S28 serine peptidase family—AoS28A and AoS28B—were found in the acidic culture medium of the mold fungus *A. oryzae* when soy proteins or wheat gliadins were used as the sole source of nitrogen [84]. AoS28A is an orthologue of the previously characterized PEP from *A. fumigatus* (AfuS28) and *A. niger* (AN-PEP). AoS28A was active in the pH range 2.0–6.0 with an optimum at pH 4.0 and using the substrate Ala-Ala-Pro-pNA. AoS28B was active in the pH range 2.0–7.0 with an optimum at pH 4.5. Both peptidases effectively degraded the immunotoxic proline-rich 33-mer gliadin peptide, especially in acidic conditions. Both enzymes produced similar but not identical patterns of peptide fragments, with different kinetics and some difference in individual hydrolysis products. All peptides larger than nine amino acid residues were completely digested by a combination of both enzymes for 20 min in acidic conditions, corresponding to conditions in the human stomach. Approximately 30–60 min were required for similar digestion when only one enzyme was used under the same experimental conditions, and AoS28B hydrolyzed the peptide more efficiently. Thus, the combination of both peptidases is important for the development of an effective oral preparation in enzyme therapy for patients with gluten intolerance.

The hydrolysis of peptides and some food proteins (in particular, gluten) by aminopeptidase and X-prolyldipeptidylpeptidase from *A. oryzae* also was investigated [85,86]. A non-specific aminopeptidase from *A. oryzae* was able to cleave the N-terminal glycine and proline residues with high efficiency, and X-prolyldipeptidylpeptidase hydrolyzed, with the specificity of DPP 4, substrates that contain X-Pro at the N-terminus. The two peptidases were synergistic and effectively hydrolyzed proline-containing peptides.

A mixture of two commercially available food-grade enzymes, aspergilopepsin from *A. niger* (ASP) and DPP 4 from *A. oryzae*, each widely used in the preparation of food, food additives, and feed, were evaluated for their use in detoxifying gluten [87]. The activity of ASP was tested against recombinant gluten proteins and synthetic gluten peptides. ASP, in contrast to mammalian pepsin, was able to hydrolyze gluten to mostly short peptides. However, unlike glutenases, such as PEP or EP-B2 of barley, which have high specificity for immunotoxic Pro- and Gln-rich gluten peptides, ASP does not have such specificity. While it can hydrolyze these peptides, the presence of competing substrates significantly slows down the hydrolysis. MS-MS analysis showed that ASP cleaves VQWPQ↓QQP↓V↓PQPHQPF of γ-gliadin, and also PFSQ↓Q↓Q↓QPV of glutenin. In the absence of other protein substrates, ASP cleaved the 33-mer peptide LQLQPFPQPQLPYPQPQLPYPQPQPQF from α2-gliadin, as well as the 28-mer peptide PFPQPQLPYPQPQLPYPQPQLPYPQPQP (a shortened derivative of the 33-mer peptide). However, in the presence of more complex substrates, such as casein, ASP activity toward peptides containing immunotoxic epitopes was slower. Although each enzyme was unable to detoxify gluten under simulated gastric conditions (0.01 M HCl was added periodically for an hour), a combination of the two enzymes led to the detoxification of moderate amounts of dietary gluten. Since these enzymes have already been proven safe for human consumption, this enzyme therapy could provide at least short-term relief of the inflammatory bowel response in patients with gluten disease and suffering from accidental gluten exposure. In addition, ASP can be added to stronger and more specific glutenases, such as EP-B2 [33,88] and some microbial/fungal PEP [74], to further enhance their therapeutic effect. Therefore, controlled clinical trials of these food enzymes are needed to assess their effects on patients suffering from gluten immunity.

### 4.3. Hydrolysis of Gluten Proteins and Their Toxic Peptides by Insect Peptidases

The digestive peptidases of the yellow mealworm, *Tenebrio molitor*, a stored product pest, are of interest because the main food proteins for this insect are prolamins. Two peptidases were isolated from a midgut extract of *T. molitor* larvae with post-proline hydrolyzing activity: PPCP1 (post-proline cleaving peptidase) and PPCP2, with molecular masses of 101 and 62 kDa, respectively [89,90]. PPCP1 was localized in the contents of the acidic anterior part of the gut with a maximum activity at pH 5.6, while PPCP2 was a tissue-soluble enzyme evenly distributed along the midgut of the larvae, with a maximum activity at pH 7.9. Inhibitory analysis indicated that both enzymes were serine peptidases. A specific inhibitor of POP, Z-Pro-prolinal, completely suppressed the activity of PPCP2 and only partially inhibited PPCP1. The study of substrate specificity demonstrated that PPCP1 preferentially hydrolyzed Z-Ala-Ala-Pro-pNA, and PPCP2 preferred a shorter peptide Z-Ala-Pro-pNA. In examining all data, PPCP2 was characterized as POP, and PPCP1 as PRCP [91]. In addition, recombinant DPP 4 of *T. molitor* was able to hydrolyze gliadins more effectively than human DPP 4, and *T. molitor* prolidase was proposed as a critical enzyme for the final stages of gliadins digestion, providing hydrolysis of imidodipeptides [92,93,94].

Proteolytic activities hydrolyzing the substrate Z-Ala-Ala-Gln-pNA after a glutamine residue were detected in the extract of the midgut of *T. molitor* larvae [29]. There was one post-glutamine cleaving peptidase (PGP2AM) in the anterior part of the midgut, and two (PGP1PM and PGP2PM) in the posterior part. All the isolated enzymes hydrolyzed Z-Ala-Ala-Gln-pNA only in the presence of dithiothreitol (DTT) and were completely inactivated by E-64 (L-trans-epoxysuccinyl-leucylamido-(4-guanidino)-butane), which indicates that they are cysteine peptidases. An electrophoretic study of the dynamics of the hydrolysis of gliadins by PGP2AM and serine digestive peptidases showed that a cysteine peptidase with PGP activity contributed significantly to their hydrolysis. Similarly, PGP activity of cysteine digestive peptidases with the selective substrate Glp-Phe-Gln-pNA was also found in the midgut of *Tribolium castaneum*, a related stored product pest also from the Tenebrionidae family [30].

The efficiency of using individual PSPs is low because most are exopeptidases and cannot provide complete cleavage of gliadins. However, in the review [95], the combination of *T. molitor* digestive enzymes, mainly cysteine cathepsin L and PSPs, are proposed to degrade gliadins in cooperation. 

PSPs were found in extracts from the lesser grain borer, *Rhyzopertha dominica* [96]. An isolated but uncharacterized enzyme was effective in the hydrolysis of CD-related peptides of wheat and barley. The overall data suggests much can be learned through the study of insects that have become adapted to efficiently process cereal grains.

### 4.4. Hydrolysis of Gluten Proteins and Their Toxic Peptides by Plant Peptidases

In plants, peptidases with PGP activity were the most active in the hydrolysis of gluten proteins. A papain-like cysteine peptidase EP-B2 (EndoPeptidase from Barley) was purified and characterized from grains of sprouted barley, *Hordeum vulgare*, and the recombinant proenzyme was expressed in *E. coli* [33,88]. The zymogen (proEP-B2) was rapidly autoactivated under acidic conditions at a rate independent of the proEP-B2 concentration, yielding a mature enzyme with a molecular mass of 27.7 kDa. The ability of the enzyme to hydrolyze toxic gliadin peptides was evaluated with the 33-mer peptide LQLQPFPQPQLPYPQPQLPYPQPQLPYPQPQP with a ratio of 1:10 (enzyme:substrate) at pH 3 for 60 min (zymogen activation conditions). The analysis of the mass spectrum indicated that EP-B2 hydrolyzed bonds formed by glutamine residues LQ↓LQPFPQPQ↓LLPYPQPQ↓LPYPQPQ↓LPYPQPQ.

The effect of EP-B2 on intact toxic peptides was studied in mice whose diet contained gluten. In the stomach and intestines of control mice, a number of toxic peptides, including 11-mer PFPQPQLPYPQ, 14-mer PQPQLPYPQPQLPY, 28-mer PFPQPQLPYPQPQLPYPQPQLPQPQP, and 33-mer LQLQPFPQPQLPYPQPQLPYPQPQLPYPQPQPF, were detected by HPLC [97]. After EP-B2 was added to the mice’s food, the peptide content decreased depending on the time and concentration of the enzyme. Estimates were that the concentration of the 33-mer peptide in the stomach could be reduced by more than 50-fold. EP-B2 was resistant to the action of pepsin, but lost activity in the presence of trypsin, and it was concluded that the potential for its use is limited to the gastric phase of digestion. Moreover, the activation of proEP-B2 occurs under acidic conditions and only at pH values less than 4.0. Thus, EP-B2 should remain active during the digestion of gluten in the stomach, but will quickly break down in the duodenum. For therapeutic use, the intestinal stability of recombinant EP-B2 can be increased through modification of trypsin-sensitive cleavage sites.

**Figure 2:**
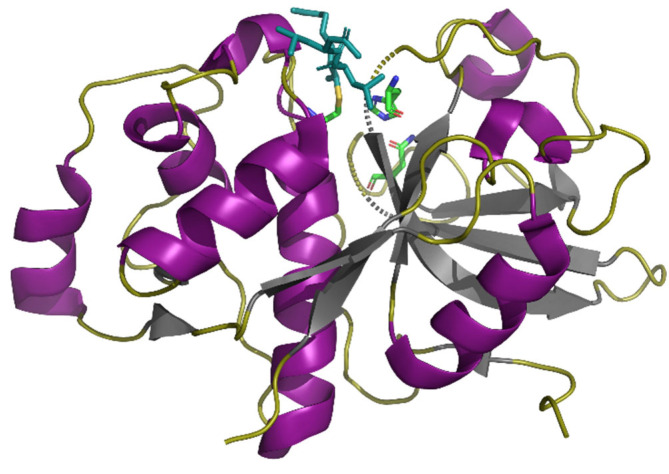
Structure of EP-B2 with the cysteine peptidase inhibitor leupeptin (PDB 2FO5) with a resolution of 2.2 Å. The α-helices of the L domain are purple, β-sheets of the R domain are gray. Amino acid residues of the active center are shown as sticks and colored by elements (C—green, N—blue, O—red). Leupeptin is shown as sticks and is teal. Structure is visualized using PyMOL (https://pymol.org/2/, accessed on 25 May 2021) (The PyMOL Molecular Graphics System, Version 1.2r3pre, Schrödinger, LLC).

X-ray diffraction analysis revealed the structure of EP-B2 with the cysteine peptidase inhibitor leupeptin (N-acetyl-Leu-Leu-argininal) with a resolution of 2.2 Å (Figure 2) [33]. The enzyme molecule consists of two domains—L (left) and R (right), with the active center located in the cavity. The L domain consists of α-helices, while the R domain consists mainly of antiparallel β-sheets and an N-terminal loop. The tertiary structure of the enzyme is stabilized by three disulfide bonds: Cys25-Cys67, Cys59-Cys100, and Cys161-Cys213. The catalytic triad is formed by the amino acid residues Asn188, His167, and Cys28. The inhibitor binds to the S1 and S3 subsites, which are necessary for substrate binding.

With enzyme therapy of CD, the use of the zymogen form of EP-B2 was proposed, from which the active EP-B2 is quickly formed in the stomach. In addition, EP-B2, together with the recombinant SC-POP from *S. capsulata* described in Section 4.1, can synergistically and completely destroy gluten [97]. With this research, ALV003 (Alvine, USA, patent WO2014116871A1), now known as the previously described latiglutenase, was developed for oral CD therapy and successfully passed phase II clinical trials [68,98,99].

The hydrolysis of gliadins under the action of cysteine peptidases from sprouted wheat seeds *Triticum aestivum* triticain-α (Triticain-α) was also studied [34,100,101]. A form of the truncated enzyme Triticain-α, lacking a signal sequence and a granulin domain (Triticain-α-GM), was recombinantly expressed in the bacterial system of *E. coli*, and hydrolyzed gliadins in a wide pH range from 3.0 to 6.5. The effect of Triticain-α-GM on the 33-mer toxic peptide from α-gliadin, formed by the cleavage of gliadins by pepsin, demonstrated the ability of Triticain-α-GM to detoxify gliadin peptides that induce an immune response in celiac patients.

Triticain-α-GM was incubated with the main gastrointestinal peptidases pepsin and trypsin, at acidic and slightly alkaline pH values, respectively. The enzyme was relatively resistant to pepsin cleavage when incubated at pH 3.0 at 37 °C for 30 min and maintained its maximum glutenase activity under these conditions. In contrast, incubation of Triticain-α-GM in the presence of trypsin at pH 8.0 and 37 °C resulted in complete degradation within 15 min. Thus, Triticain-α-GM is stable under conditions of gastric digestion, and has a significant ability to break down gluten [34].

Degradation of gliadins with high efficiency was also observed using peptidases of raw papaya milky juice [102]. The test mixture was superior to individual plant enzymes and fungal peptidases. The high activity of the mixture of enzymes from papaya latex was primarily due to caricaine, the most alkaline among the cysteine peptidases of latex. When raw papaya milky juice was added to swine gut extract, a synergistic effect was observed, with an increase in the degree of cleavage of toxic peptides 11–19 (QNPSQQQPQ) and 75–86 (RPQQPYQPQPQP) from α-gliadins. The synergistic effect was proposed to be due to a difference in the specificity of the enzymes, that is, the hydrolysis products of one enzyme can act as substrates for the second. These enzymes were used in the production of bread with a low gluten content, treating the kneaded baking dough with caricaine isolated from papaya latex extract [103,104]. The gluten level in the dough treated with purified enzyme was significantly lower compared to the crude extract treatment. As for the quality, bread made from caricaine-treated dough turned out to be looser and more porous, with a harder and darker crust, compared to the control sample. The bread obtained from the dough fermented for 7 h at 37 °C was considered the most optimal in terms of quality [103].

Thus, plant cysteine peptidases, along with bacterial and fungal peptidases, can be considered as candidates for use in the production of gluten-free products and in the treatment of CD patients. However, more clinical data on the use of glutenase preparations as a CD therapy will be necessary to validate effective treatments in gluten-dependent disorders (Table 3). 

It should be noted that the DPP 4 enzyme often used in clinical testing is not active at low pH and, therefore, is not very effective in the acidic environment of the stomach. In addition, DPP4 has no endopeptidase activity and, in the absence of other enzymes, can only act on the N-terminus of proteins, so large immunogenic fragments of gluten can remain uncleaved [109]. The role of gliadins in people with non-celiac gluten sensitivity (NCGS) remains unknown, and a rationale for the use of gluten-hydrolyzing enzymes in such people is unclear [110]. Immuno-mediated enteropathy, CD or gluten disease, and NCGS are two different clinical conditions, although both are caused by wheat gliadin. People with NCGS suffer from the same symptoms as patients with CD (diarrhea, stomach problems, fatigue, joint pain, etc.), but without the histological and serological markers characteristic of CD [111]. In patients with NCGS, gluten consumption also can lead to potentially harmful long-term effects [112]. A significant increase in markers of intestinal epithelial damage and systemic immune activation was found in this group of patients receiving food containing cereal proteins. In contrast to CD, the observed humoral immune response with NCGS to gluten does not depend on the enzymatic activity of TG2 and the presence of HLA-DQ2/DQ8, so hydrolysis products are assumed to target certain epitopes other than those in CD. Although NCGS individuals showed increased levels of antibodies against native gliadin, the role of gluten in this condition is debated. While some studies have shown that gluten consumption leads to gastrointestinal symptoms in people with NCGS [113,114], others have not confirmed the association [115]. The latest study indicated that bread pre-processed with AN-PEP had a 40% reduction in gluten [116]. However, eating this bread did not reduce the symptoms of gluten sensitivity in people with NCGS. The results could be due to an insufficient reduction in the gluten content, but instead of gluten, other compounds present in wheat or related cereals may be involved in the onset of symptoms. The later hypothesis is supported by the results of Skodje et al. [117], where a randomized double-blind study using granola bars demonstrated that in NCGS people, symptoms were caused mainly by fructans, not gluten. 

## 5. Conclusions

Hydrolysis of proline/glutamine-rich proteins is difficult because most broad-spectrum peptidases are unable to cleave the peptide bonds formed by proline and glutamine residues. However, proline/glutamine-rich proteins such as prolamins become pathogenic under certain physiological conditions, but their proteolysis can provide a therapeutic effect. Thus, prolamins and their immunogenic peptides in human food cause an autoimmune response in predisposed people, leading to the development of CD. This review summarized the use of various PSPs for the hydrolysis of these proteins. Among them, the greatest attention is paid to the study of POP and PEP, since these peptidases hydrolyze long protein sequences into shorter fragments. However, a sufficiently complete hydrolysis was possible only with the combined use of several different PSPs. Promising results are found in studies of mixed complexes of PSPs and subtilisin-like or cysteine peptidases with PGP activity.

With that, a number of questions remain unanswered or insufficiently studied related to the effective use of peptidases to reduce the toxic effects of prolamins and their immunogenic peptides. In addition, it is necessary to evaluate whether the enzymatic pretreatment of wheat flour and the removal of harmful components for CD may lead to the loss of characteristics that make gluten-containing products preferable for food production. Before incorporating commercially available enzyme preparations to reduce gluten sensitivity, such as those containing various glutenases derived from bacteria or fungi, it is important to gather the available scientific data on their effectiveness and safety. 

The use of these enzymes cannot be recommended to compensate for the intake of large quantities of gluten (consumed unintentionally or intentionally). Despite the fact that their effectiveness can be quite high, even a small amount of gluten or its peptides that reach the duodenum can be harmful to CD patients. In addition, the effectiveness of the enzymes in vitro is affected by the composition of the food, and this effect has not yet been properly investigated in vivo. 

The available biochemical data on a particular enzyme may help to select a promising candidate for possible enzyme preparations, but further clinical trials are needed to confirm the therapeutic effectiveness of the selected enzyme in the treatment of gluten intolerance. So far, an analysis of the results suggests that enzyme therapy alone is not sufficient for the treatment of CD. Such therapy is probably not able to neutralize the large amount of gluten present on average in the human diet based on wheat or similar cereals. However, enzyme therapy can reduce the gluten-induced effects observed in the background of GFD practices that occur by the unintentional consumption of small amounts of gluten, that is, to act as a pharmacological supplement to GFD.

## Figures and Tables

**Table 1:** Peptide sequences of wheat gliadins that are resistant to proteolysis.

Size	Peptide Sequence	Origin	Position	Composition (Pro, Gln), %
33-mer	LQLQPFPQPQLPYPQPQLPYPQPQLPYPQPQPF	α-2 gliadin	56–88	Pro 40, Gln 30
26-mer	FLQPQQPFPQQPQQPYPQQPQQPFPQ	γ-5 gliadin	26–51	Pro 35, Gln 46
20-mer	LQPQQPFPQQPQQPYPQQPQ	γ-5 gliadin	60–79	Pro 35, Gln 50
20-mer	QQQQPPFSQQQQSPFSQQQQ	glutenin		Pro 15, Gln 60
19-mer	LGQQQPFPPQQPYPQPQPF	α-gliadin	31–49	Pro 37, Gln 37
17-mer	QLQPFPQPELPYPQPQS	α-gliadin	57–73	Pro 35, Gln 29
15-mer	VQGQGIIQPQQPAQL	γ-gliadin		Pro 13, Gln 40
15-mer	QQPPFSQQQQQPLPQ	glutenin		Pro 27, Gln 53
14-mer	PQPQLPYPQPQLPY	α-2 gliadin	62–75	Pro 43, Gln 29
13-mer	LGQQQPFPPQQPY	α-gliadin	31–43	Pro 31, Gln 38
12-mer	FSQPQQQFPQPQ	γ-5 gliadin	102–113	Pro 25, Gln 50
12-mer	QLQPFPQPQLPY	α-9 gliadin	57–68	Pro 33, Gln 33
10-mer	QPQQSFPQQQ	γ-gliadin		Pro 20, Gln 60

**Table 2:** Specificity of proline-specific peptidases.

Number	Peptidase Class	Enzymes	Substrates ^1^
1	Serine peptidases	Prolyloligopeptidase (POP), prolylendopeptidase (РЕР), fibroblast activation protein (FAP)	(Xaa)*n*-Xbb-Pro↓Xbb-(Xaa)*_n_*, *n* = 1–13 (the length of the peptide is approximately 30 amino acid residues)
2		Dipeptidylpeptidases (DPP) 2, DPP 4, DPP 8, DPP 9, FAP	Xbb-Pro↓Xbb-(Xaa)*_n_*, *n* = 2–12
3		Prolylcarboxypeptidase (PRCP)	(Xaa)*_n_*-Xbb-Pro↓Xbb, *n*—any number
4	Metallopeptidases	Aminopeptidases P (APP) 1, APP2, APP3	Xbb↓Pro(Xaa)*_n_*, *n* = 1–9
5		Prolidase	Xbb↓Pro

^1^ Xaa—any amino acid; Xbb—any amino acid, except Pro.

**Table 3:** Current data on the drugs for enzyme therapy of gluten sensitivity.

Product	Company	Base of the Drug (Origin)	Clinical Trial Phase	References
KumaMax, Kuma030, Kuma062, TAK-062	Takeda Pharmaceutical Company Limited, Tokyo, Japan	Kumamolysine-As *Alicyclobacillus sendaiensis*	1	[66,105,106]
Latiglutenase ALV003	Alvine Pharmaceuticals Inc., San Carlos, CA, USA	Prolyl oligopeptidase (POP) *Sphingomonas capsule* + cysteine peptidase from barley (EP-B2)	2	[67,68,69,71,73]
Tolerase G	DSM Nutritional Products, Kaiseraugst, Switzerland	Prolylendopeptidase of the mold fungus *Aspergillus niger* (AN-PEP)	Dietary supplement	[77,81]
AMYRA’s enzymes, AMY01	AMYRA Biotech AGBasel, Switzerland	Combination of fungal exopeptidases	Dietary supplement	[107]
AMYRA’s enzymes, AMY02	AMYRA Biotech AGBasel, Switzerland	Combination of fungal exopeptidases	Pre-clinical	[107]
Nemysis E40	Nemysis Ltd., Dublin, Ireland	Endopeptidase soil Actinoallomurus strain	Pre-clinical	[108]

## Data Availability

Not applicable.
